# Development of Broad-Range Microbial Minimal Culture Medium for Lanthanide Studies

**DOI:** 10.3390/microorganisms12081531

**Published:** 2024-07-26

**Authors:** Gianmaria Oliva, Giovanni Vigliotta, Luca Di Stasio, Ermanno Vasca, Stefano Castiglione

**Affiliations:** Department of Chemistry and Biology “A. Zambelli”, University of Salerno, 84084 Fisciano, SA, Italy; gioliva@unisa.it (G.O.); ldistasio@unisa.it (L.D.S.); evasca@unisa.it (E.V.); scastiglione@unisa.it (S.C.)

**Keywords:** REE, *Bacillus stratosphericus*, lanthanide toxicity, environmental screening, complexation equilibria, cerium, lanthanide accumulation, *Debaryomyces hansenii*

## Abstract

Rare Earth Elements (REE), also known as Lanthanides (Ln^3+^), are a group of 17 elements showing peculiar physical and chemical properties. Unlike technological applications, very little is known about the physiological role and toxicity of Ln^3+^ on biological systems, in particular on microorganisms (e.g., bacteria), which represent the most abundant domains on our planet. Up to now, very limited studies have been conducted due to Ln^3+^ precipitation with some anions commonly present in the culture media. Therefore, the development of a minimal medium is essential to allow the study of Ln^3+^-microbial interactions, limiting considerably the precipitation of insoluble salts. In this regard, a new minimal culture medium capable of solubilizing large amounts of Ln^3+^ and allowing the growth of different microbial taxa was successfully developed. Assays have shown that the medium is capable of solubilizing Ln^3+^ up to 100 times more than other common culture media and allowing the growth of 63 bacteria and 5 fungi. The kinetic growth of one yeast and one Gram-positive bacterium has been defined, proving to support superior growth and biomass compared to other commonly used minimal media. Moreover, the sensitivity and uptake/absorption of a *Bacillus stratosphericus* strain were tested, highlighting its capability to tolerate concentrations up to 10 mM of either Cerium, Gadolinium or Lanthanum and accumulate different quantities of the three.

## 1. Introduction

Lanthanides (Ln^3+^), also commonly known as Rare Earth Elements (REE), represent a family of 17 chemical elements located in the f block of the element periodic table. Their versatility and unique physical and chemical properties make them valuable elements for a wide range of applications, contributing to technological and industrial innovations [1]. One of the most peculiar Ln^3+^ characteristics is the ability to emit visible light when excited, producing brilliant and luminescent colors. This property is exploited for the production of fluorescent displays, such as those present in liquid crystal televisions (LCDs), flat-panel monitors [2] and smartphones [3]. Ln^3+^ are also key components of ferromagnetic materials employed for solid-state memories and the production of high-power permanent magnets [4]. Some elements, such as europium (Eu) and terbium (Tb), are used in the production of energy in compact fluorescent (CFL) and energy-saving lamps [5]; others, such as yttrium (Y) and neodymium (Nd), are used to create active components in lasers [6]. Cerium (Ce), the most widely used Ln^3+^, is employed as a key component in exhaust automotive catalysts, to reduce harmful emissions [7]. Finally, elements such as gadolinium (Gd) are widely used to produce electronic device materials [8].

Despite the widespread use of these elements, the availability of Ln^3+^ is scarce due to a limited distribution of reserves on Earth and geopolitical and economic issues that often play a key role in their supply [9]. Furthermore, their extraction is expensive and harmful to the environment, making investments in the search for new mining sites unattractive [10,11]. About that, many countries are trying to diversify their Ln^3+^ supply sources, develop new technologies for their recycling, and recover from electronic waste [12,13].

Unlike technological applications, very little is known about the role of Ln^3+^ in biological systems and their physiological or toxic effects on different organisms. Some studies have reported that plants cultivated in the presence of low Ln^3+^ doses improved root development and plant growth [14,15]. On the contrary, exposure to high doses led to the formation of reactive oxygen species (ROS), biochemical and molecular alterations, and growth reduction [14,16]. In studies on aquatic invertebrates (*Sphaerechinus granularis* and *Arbacia lixula)*, exposure to Ln^3+^ was associated with damage to the nervous or excretory systems other than cytogenetic anomalies [17]. Moreover, in humans, a correlation was observed between some diseases (e.g., endomyocardial fibrosis, brain tumor) and the exposure to La^3+^, Ce^3+^, and Gd^3+^ or their accumulation in tissues [18,19]. Regarding microorganisms, more information is available for methylotrophic bacteria, where a biological role was evidenced for Ln^3+^ in the catalysis of certain pyrroloquinoline quinone (PQQ)-dependent alcohol dehydrogenases (ADHs, MDHs) [20,21]. Other studies have investigated the transport, accumulation, and storage of Ln^3+^ in methanotrophs, highlighting the existence of periplasmic proteins with high affinity for Ln^3+^ [22]. Lanmodulin (LanM), a periplasmic protein with a high affinity for Sm^3+^, Nd^3+^, and La^3+^, has been identified in *Methylorubrum extorquens*, and it was hypothesized its use in enzymatic functions [23]. Likewise, Lanpepsy (LanP), another periplasmic protein involved in the lanthanide response, was identified in *Methylobacillus flagellates* [24].

Therefore, the importance and enormous diffusion of Ln^3+^ in human activities pose several problems, including the elucidation of their biological role and effects on the biosphere, the definition of strategies to reduce their environmental impact, and the development of technologies for their recovery and recycling.

The aim of our research is to contribute to the improvement of knowledge on Ln^3+^ in the field of microbiology through the formulation of a suitable non-selective minimal culture medium for the study of a wide range of microorganisms. The use of minimal media is essential for physiological, metabolic, molecular, and genetic studies because it allows the control of experimental conditions. Unlike other culture media, minimal ones do not include complex ingredients in their composition (e.g., peptones, extracts, casamino acids), avoiding or limiting the presence of non-quantifiable and/or unwanted macro- and micro-nutrients and growth factors.

To date, one of the main limitations in Ln^3+^ research is due to poor solubility and consequent precipitation at low concentrations in mineral solution, caused by the reaction with phosphate, sulfate, or carbonate ions [25,26]. These precipitates can agglomerate and deposit as pellets, including the microorganisms themselves, interfering with microbial growth and biological activities by different mechanisms, such as: (i) reducing the bioavailability of nutrients (i.e., phosphorus, nitrogen); (ii) limiting the binding sites involved in the absorption of the latter, by accumulating on the microbial surface and generating a physical barrier [27]; (iii) affecting the chemical environment (i.e., pH and ion concentrations) and interfering with the biochemical and metabolic reactions [28]; (iv) generating agglomerates which, by adhering to the microbial surface, prevent movement and interactions with other cells or the substrate [29].

For the above reasons, it is essential to formulate a culture medium that minimizes the formation of insoluble compounds, reducing their interference. To avoid the precipitation of Ln^3+^, the use of organic chelators such as EDTA, EDDS, DTPA, and TTHA is reported in the scientific literature [30]. However, it has been demonstrated that many of these compounds are toxic for microorganisms since they interfere with their metabolism [31,32]. Consequently, the addition of a suitable chelator could be a valuable system to overcome this obstacle.

As a contribution to this topic, we report here the formulation of a minimal culture medium based on citrate buffer and a reduced concentration of phosphate, allowing the solubility of Ln^3+^ to be increased up to a hundred times compared to those obtained in other common minimal media. Citrate acts as a chelator that complexes Ln^3+^, preventing their precipitation [33]; furthermore, being an organic source of carbon and a growth factor for many microorganisms [34], it is not toxic for most of them.

## 2. Materials and Methods

### 2.1. Preparation and Use of Minimal Culture Medium for Lanthanides

The Minimal Culture Medium for Lanthanides (MCML) was prepared by dissolving in 1.0 L of distilled water: 2.0 g NH_4_SO_4_, 0.2 g MgSO_4_·7H_2_O, 0.4 g KH_2_PO_4_, 10.02 g sodium citrate dihydrate (Na_3_citOH·2H_2_O; here and in the following the trinegative citrate ion will be indicated as citOH^3−^), 0.94 g citric acid (H_3_citOH), 2.0 g D-glucose, 0.0001 g FeSO_4_·7H_2_O, 100 µL micronutrient solution (stock solution was prepared dissolving in 100 mL of distilled water: 10.0 mg H_3_BO_3_, 11.19 mg MnSO_4_ H_2_O, 124.6 mg ZnSO_4_·7H_2_O, 78.22 mg CuSO_4_·5H_2_O, 10 mg Na_2_MoO_4_·2H_2_O) and pH was adjusted at 6.0 with a few drops of NaOH (2.0 M). The medium was sterilized by autoclaving for 15 min at 120 °C.

### 2.2. Complexation Equilibria Model

A complexation equilibria model was elaborated in PyES (Python Equilibrium Species) software, version v1.1.3 [35], considering the complex formation between Ce^3+^, citrate and phosphate at different pH values. For the model elaboration, the total concentration of Ce^3+^ was assumed to be 1.0 mM, while the analytical phosphate and citrate concentrations were the same as reported in MCML (3.0 mM and 40.0 mM, respectively). The complexation constants and reactions considered are described in Table S1.

### 2.3. Solubility Test

Initially, a Cerium (Ce) solubility test was conducted in MCML using increasing concentrations of Ce(NO_3_)_3_·6H_2_O (0.01, 0.05, 0.1, 0.5, 1.0, 2.0, 3.0, 5.0 mM) at different pHs (ranging between 2.0 and 8.0). To verify if any precipitates were to form, Ce^3+^ was added to MCML at increasing concentrations (as described above) and then placed in transparent tubes to visualize any precipitates. After 24 h, in some cases, a white precipitate at the bottom of the tube could be observed, highlighting the Ln^3+^ insolubility at the assayed pH and Ce^3+^ concentration.

Afterward, we also conducted other solubility tests either in MCML or in other three commonly used minimal culture media (Table 1): Davis and Mingioli (DM) [36] and Dworkin and Foster (DF) [37], supporting bacterial growth, and Czapek Dox (CD) [38] for fungi growth. The solubility of three Ln^3+^: Cerium (Ce), Lanthanum (La), and Gadolinium (Gd), was evaluated as described above by dissolving, separately, increasing concentrations (0.01, 0.05, 0.1, 0.5, 1.0, 2.0 mM) of each Ln^3+^. In particular, Ce(NO_3_)_3_·6H_2_O, La(NO_3_)_3_·6H_2_O, and Gd(NO_3_)_3_·6H_2_O were dissolved in: MCML, DM, CD and DF. After 24 h, a fluffy white precipitate at the bottom of the tube highlighted the Ln^3+^ insolubility at the assayed concentrations. Moreover, the possible presence of colloidal precipitates in the culture media was verified using turbidimetry (Turbidimeter model TU5300sc by HACH, Loveland, CO, USA). The concentration of Ln^3+^ in the homogeneous solutions was verified using ICP-MS (iCAP RQplus ICP-MS, by ThermoFisher, Monza, Italy) and compared with the analytical concentration. The results agreed within ±1%.

### 2.4. MCML Potentiality for the Cultivation of Different Microorganisms

MCML agar plates were prepared to evaluate its capability to allow the cultivation of different microbial species. Solid MCML was prepared by adding 15.0 g L^−1^ of agar, and autoclaved for 15 min at 120 °C. The results on the growth were compared with the following agarized (15.0 g L^−1^ agar) DM, CD (either with sucrose 30.0 g L^−1^, or 2.0 g L^−1^), DF and with the Plate Count Agar (PCA) rich medium (to 1.0 L of PCA: 5.0 g tryptone, 2.5 g yeast extract, 1.0 g glucose were added). A reduced amount of sucrose was used in CD to compare the bacterial growth with that obtained in the case of other tested culture media, if the carbon source was glucose (2.0 g L^−1^). For this purpose, 89 different microorganisms were used. Among these, 82 were bacterial strains of environmental origin (46 Gram-negative and 36 Gram-positive), two were of human health relevance (*Escherichia coli* and the pathogenic *Staphylococcus aureus* [39]), and 5 were fungi (2 yeast and 3 molds) (See Table S2). The 89 microorganisms cultivated for 24–36 h on agar plates were inoculated in 200 µL of Luria–Bertani (LB) liquid medium (to 1.0 L of medium: 10.0 g tryptone, 5.0 g yeast extract, 5.0 g NaCl, were added) in a 96-multiwell plate, slowly shaken (60 RPM) overnight at 28 °C for environmental microorganisms, with the exception of the two mesophilic bacterial strains (*E. coli* and *S. aureus*) grown at 37 °C. Then, they were spotted by means of 96-Pin Microplate Replicator on each agar medium and incubated at the corresponding temperatures.

The environmental microorganisms were isolated from both soil and rhizosphere; the two yeasts, *Debaryomyces hansenii* [40] and *Saccaromyces cerevisiae*, were from an in-house microbial collection of Prof. Giovanni Vigliotta (stored at −80 °C at the Department of Chemistry and Biology of the University of Salerno). Then, *E. coli* (strain JM 109) was purchased at Promega Italia Srl, Milan, Italy (http://www.promega.com/products, accessed on 1 June 2024; cat. no P9751), while *S. aureus* was isolated from hospital patients as part of the same collection mentioned above. The microorganism growth was evaluated at one day, two days, and five days.

### 2.5. Determination and Comparison of Growth Parameters

The growth in MCML was compared with that of two minimal media specific for bacteria and fungi, respectively, recognized by the world literature and widely used (Table 1). A *Bacillus stratosphericus* strain, previously isolated by Dr. Gianmaria Oliva [41,42], was cultivated in both MCML and DM, whilst *D. hansenii* in both MCML and CD was modified by replacing the glucose with 30.0 g L^−1^ sucrose as a carbon source because it was better used by the yeast [43].

Initially, a colony from fresh culture plates was inoculated in 3.0 mL of LB medium and incubated at 30 °C overnight. Then, the liquid culture was centrifugated at 2500 RCF for 15 min, the supernatant was discarded, and pellets were resuspended in 2.0 mL of sterilized distilled water and vortexed for 30 s.

Aliquots of bacterial/fungal suspensions were inoculated in 50.0 mL of the culture media (Table 1) in 250 mL Erlenmeyer flasks at a cellular density of 0.01 measured by optical density at 600 nm (OD_600_). The flasks were incubated at optimal growth temperatures, 42 °C for *B. stratosphericus* and 30 °C for *D. hansenii*, under constant shaking at 200 RPM. The experiment was conducted in triplicate. The microorganism growth was followed by measuring OD_600_ over time using an ONDA UV-20 spectrophotometer (Sinergica soluzioni, Milan, Italy). The absence of contamination was repeatedly checked by morphologic analysis (plate assay and microscopy).

The average growth rate (R) was calculated by determining generation number (n) at the middle of the exponential phase, with the relation: [log(N_2_ − log(N_1_)]/log2, where N_1_ and N_2_ were the number of cells at time t_1_ and time t_2_ of exponential growth, respectively. R was n/D_t (t2 − t1)_, and reported as the number of generations at hours (h^−1^) or days (d^−1^).

The dried biomass was weighed at the end of the growth, in the stationary phase. Specifically, for each culture, a volume of 5.0 mL was withdrawn and centrifuged at 6500 RCF for 15 min; the supernatant was discarded; and the pellet was dried in an oven at 70 °C to achieve a constant weight.

### 2.6. Evaluation of Ln^3+^ Toxicity and Accumulation

The toxicity of three different Ln^3+^ (Ce^3+^, Gd^3+^, La^3+^) was evaluated in the case of *B. stratosphericus* strain in agarized MCML, using two very high concentrations: 5.0 or 10.0 mM for Ce(NO_3_)_3_·6H_2_O; 5.0 or 10.0 mM for La(NO_3_)_3_·6H_2_O; 10.0 or 20.0 mM for Gd(NO_3_)_3_·6H_2_O. At first, the salts were dissolved in distilled sterile water and filtered through cellulose acetate membranes (cut-off 0.22 μm); then, the necessary Ln^3+^ amount was slowly added under constant shaking to agarized MCML (at 55 °C) before its polymerization. The *B. stratosphericus* was previously cultivated in liquid MCML overnight at its optimal growth temperature (42 °C), under constant shaking at 200 RPM, then the cell suspensions were serially diluted with fresh MCML (1:10, 1:100, 1:1000, 1:10,000, 1:100,000) and 100 µL were spread on MCML agar plates. The latter were incubated for 24 h at 42 °C, and the number (colony-forming units, CFU), size, and morphology of colonies were determined.

The capability of *B. stratosphericus* to accumulate three Ln^3+^ (Ce^3+^, La^3+^, Gd^3+^) was evaluated by growing the strain in 40 mL of MCML in Erlenmeyer flasks (250 mL) in the presence of 500 µM of each single Ln^3+^ salt. A bacterial sample without Ln^3+^ was considered a control. The flasks were prepared in duplicate and incubated for 24 h at 42 °C under constant shaking at 200 RPM. Afterwards, the cell suspensions were transferred into 50 mL tubes and centrifuged at 10,000 RCF for 15 min. The supernatant was discarded; the pelleted biomass was rinsed three times with distilled water and then dried in an oven at 70 °C for two days. Finally, the dried biomass was weighed and mineralized in 1.0 mL of nitric acid (65% *w*/*w*) by incubating at 70 °C overnight. The mineralized biomass was diluted at a ratio of 1:30 with ultrapure distilled water to reach a final nitric acid concentration of approximately 2.0%. At the end, the samples were analyzed using an ICP-OES (Optima 7000 DV, Perkin Elmer, Milan, Italy), and the three accumulated Ln^3+^ were quantified. Different concentrations from 0.01 up to 10 mg L^−1^ of rare earth element mix for ICP (Sigma-Aldrich, Milan, Italy) were used to generate a calibration standard curve (R^2^ = 0.99).

## 3. Results

### 3.1. Solubility in MCML

A minimal culture medium for lanthanides (MCML) was developed by adapting opportune concentrations of mineral macro- and micro-nutrients, citric acid and sodium citrate (0.94 g L^−1^ and 10.02 g L^−1^, respectively), as citrate buffer. The concentrations of the latter were chosen to limit microbial toxicity and to maintain buffer capability at an optimal pH ranging from 5.5 up to 6.5.

Initially, it was evaluated the MCML capability to avoid, at different pHs, precipitation of Ce, one of the most commonly used Ln^3+^. Hence, in the MCML, an increase in the solubility of Ce^3+^ was observed at the lower pH values. In fact, the maximum solubility was achieved at pH 2.0 ([Ce^3+^] = 5.0 mM), while at pH above 7.0, a precipitate was already observed at 1.0 mM Ce^3+^ (Table 2).

### 3.2. Formation of Ce Complexes

**Figure 1:**
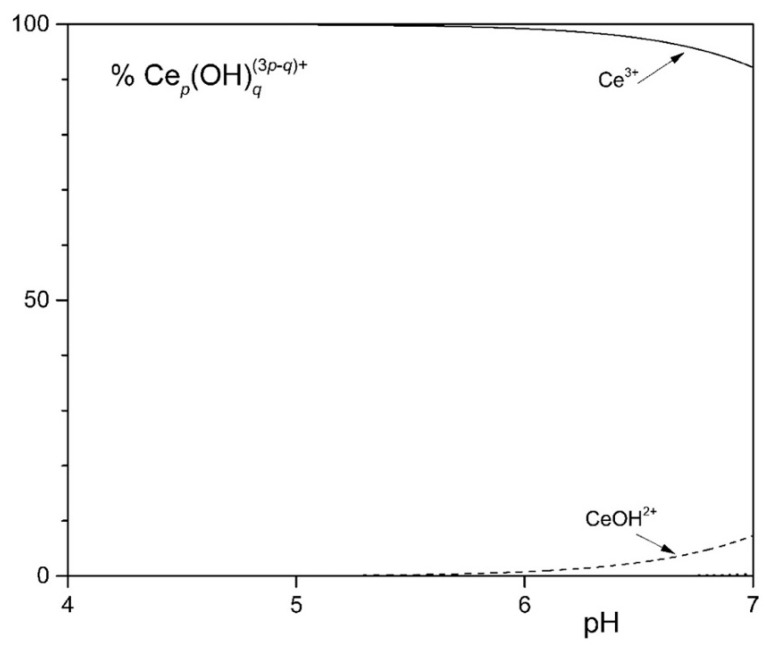
Distribution diagram of the hydrolytic species of the Ce^3+^ ion at 1.0 mM [Ce^3+^].

To study the solubility of Ln^3+^ in the MCML, a simplified model was set up that took into account complexation equilibria between Ce^3+^, phosphate, and citrate at different concentrations and pHs. The simplest model considered assumes only the hydrolyzed forms of the Ce^3+^ ion. In the 4.0 < pH < 7.0 interval (typically used to cultivate different microorganisms), more than 90.0% of the ions in solution are in the form of Ce^3+^ (Figure 1). In the second model, it was observed that in the presence of 3.0 mM of total phosphate, already at pH 1.35 and 1.0 µM Ce^3+^, a formation of solid CePO_4_(s) occurs (Figure S1). In the third model, the complexes formed between Ce^3+^ and citrate are described. In particular, at pH 6.0, the one used for microorganism cultivation in the MCML, the most abundant specie was Ce(citOH)_2_^3−^ (~100%, Figure S2). Therefore, it was hypothesized that the addition of citrate to the medium culture as a Ce-complexing agent could prevent the precipitation of solid CePO_4_(s).

**Figure 2:**
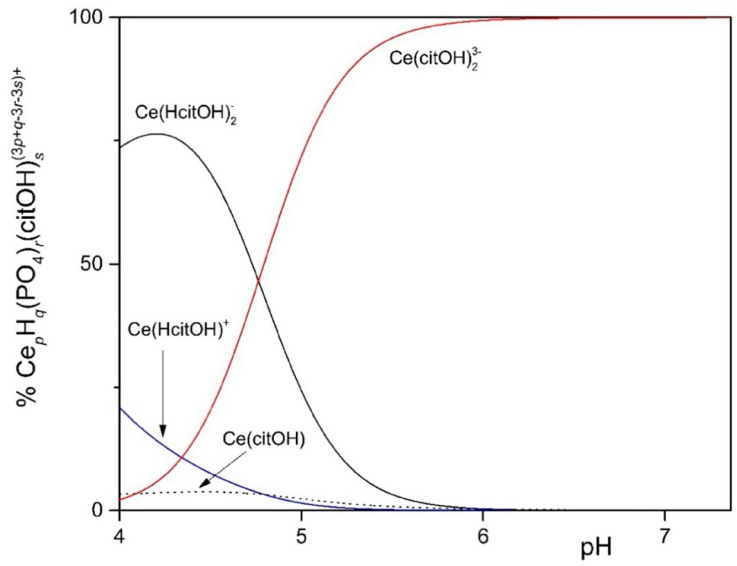
Distribution diagram of 1.0 mM Ce^3+^ in the presence of 3.0 mM total phosphate and 40.0 mM total citrate up to pH 7.36, when the formation of solid CePO_4_ is observed.

**Figure 3:**
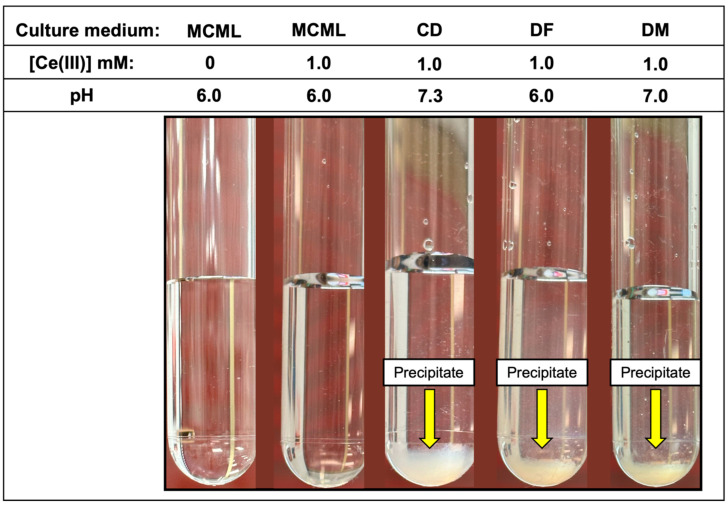
White fluffy cloud precipitate observable in the test-tubes at 1.0 mM of Ce^3+^ in CD, DF, and DM culture media.

The distribution diagram reported in Figure 2 shows Ce-complexes considering in the model 1.0 mM Ce^3+^, 3.0 mM total phosphate, and 40.0 mM total citrate. The abscissa runs up to pH 7.36 when the formation of solid CePO_4_(s) is observed. Thus, in the data set used to plot the diagram, a logK_s_ of −19 had to be assumed for CePO_4_(s) freshly formed, instead of the value of −26.27 reported for the aged solid, to model the experimental observations showing non-precipitation up to a pH of about 7.5 (Figure 3—MCML at 0 and 1.0 mM Ce^3+^).

### 3.3. Solubility of Trivalent Lanthanides in Different Culture Media

In addition to Ce^3+^, the solubility of other widespread and used Ln^3+^, such as La^3+^ and Gd^3+^, was evaluated.

In the MCML, a solubility up to 100 times greater than DF and DM (1.0 mM vs. 0.01 mM) was observed for Ce^3+^ and La^3+^, where a precipitate was already recorded at 0.05 mM (Table 3). While in CD, even at the 0.01 mM Ce^3+^ or La^3+^ concentration, a precipitate, as a white fluffy cloud, was observed (Table 3). Among the three Ln^3+^ in the test tube assay, Gd seems to be the most soluble one; in fact, in MCML, no precipitate was observed up to 5.0 mM, whilst for both DF and DM, a solubility no higher than 0.05 mM was recorded. Once again, in CD, even at a Gd^3+^ concentration of 0.01 mM, a precipitate was observed (Table 3). Finally, the precipitate was observed at the highest Ce^3+^ concentration in the various tested culture media (Figure 3).

### 3.4. Evaluation of MCML Suitability for Microorganism Growth

**Figure 4:**
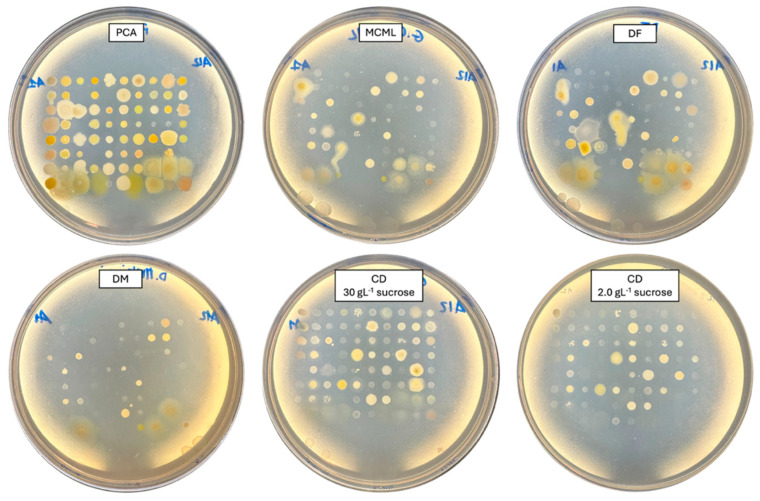
Bacterial cultivation on different agarized culture media after 48 h of incubation.

**Figure 5:**
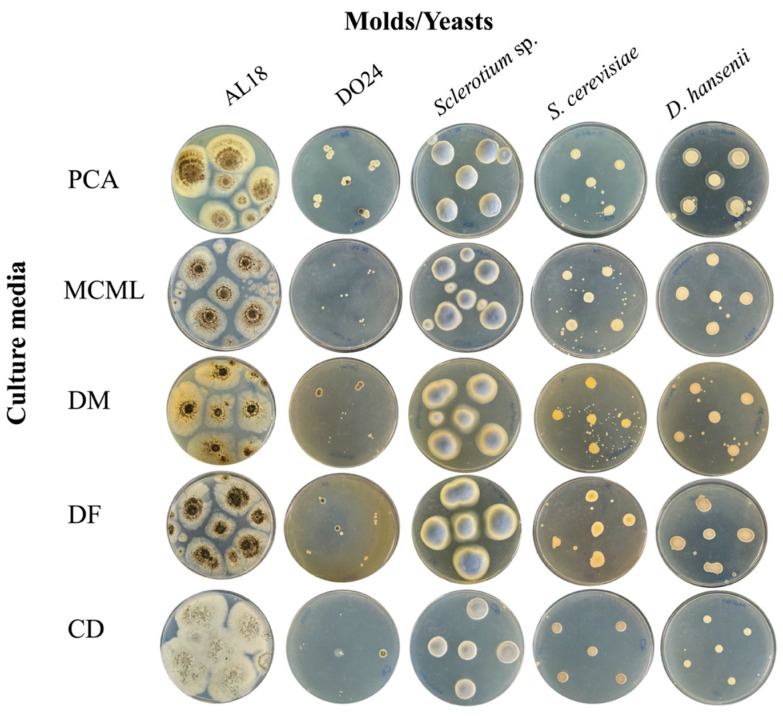
Fungi cultivation and growth on different agarized culture media after 5 days of incubation.

The potential of MCML on different microorganism growth was evaluated by testing a panel of 84 bacteria (Figure 4, Table S2, Figure S3) and five fungi (two yeasts and three molds) (Figure 5, Table S2). We used the PCA as a control medium, where all microorganisms were able to grow. It was observed that MCML supported the growth of 75% (63 of 84), whilst DF saw a growth of 81% (68 of 84) (Table S2). The DM culture medium allowed the cultivation of only 50% (68 of 84) of the total tested bacteria (Table S2). Finally, the CD medium supported more than 90% of microbial growth for both amounts of used sucrose (81 of 84 with 30.0 g L^−1^ sucrose and 78 of 84 with 2.0 g L^−1^) (Table S2). Finally, all tested fungi (AL18, DO24, *Sclerotium* sp., *D. hansenii*, and *S. cerevisiae*) grew on all the tested culture media (Figure 5, Table S2).

### 3.5. Evaluation of MCML Medium Growth Parameters

**Figure 6:**
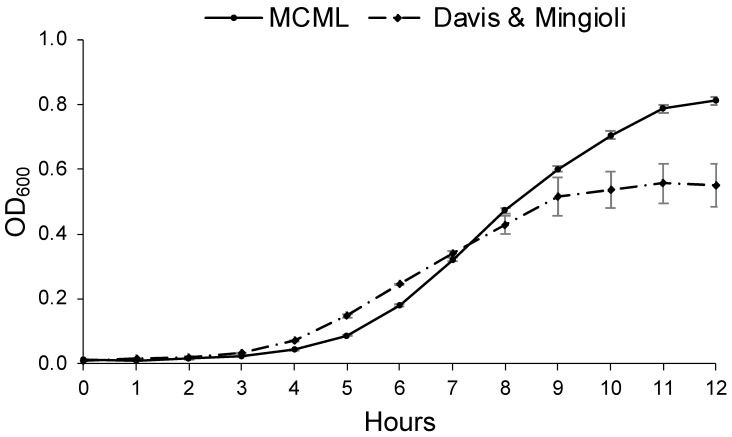
Growth curve of *B. stratosphericus* in MCML and Davis and Mingioli (DM) media.

Growth parameters for environmental bacteria and fungi were evaluated in MCML and compared to a reference minimal medium. For this purpose, we considered *B. stratosphericus* and *D. hansenii* to be the most extensively characterized microorganisms among those examined [40,41,42]. Figure 6 illustrates *B. stratosphericus* baseline growth curves cultivated in two different media: MCML and DM. The growth parameters (cell density at the stationary phase, lag phase duration, growth rate) for all baseline experiments were collected and evaluated. It was observed that the lag phase was slightly shorter in DM than in MCML medium, <1.0 h and ≥1.0 h, respectively. The average growth rate was greater in MCML (0.7 h^−1^) compared to DM (0.6 h^−1^). The final biomass measured in the stationary phase was almost twice as much in MCML (0.81 ± 0.01 OD_600_, which means 5.0 ± 0.2 mg mL^−1^) compared to DM (0.55 ± 0.07 OD_600_, 2.5 ± 0.7 mg mL^−1^).

**Figure 7:**
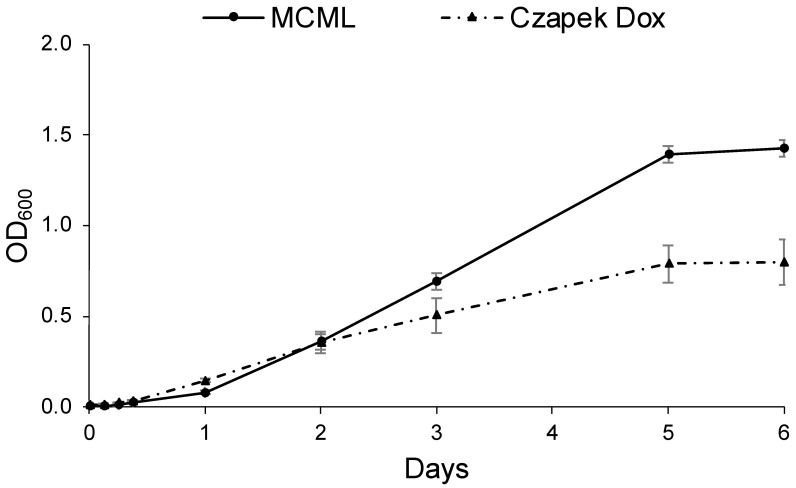
Growth curve of *D. hansenii* in MCML and Czapek Dox (CD) media.

Likewise, Figure 7 shows the *D. hansenii* baseline growth curves cultivated in MCML (replacing the carbon source with sucrose 30.0 g L^−1^) and in the most yeast-specific CD; it was observed that the lag phase was <3.0 h in CD, while it was >3.0 h in MCML. The average growth rate was greater in MCML (0.65 d^−1^) compared to CD medium (0.49 d^−1^). Again, approximately two times greater total biomass was obtained in MCML compared to CD medium (1.42 ± 0.05 OD_600_ vs. 0.80 ± 0.13 OD_600_; 8.5 ± 0.1 mg mL^−1^ vs. 4.1 ± 0.7 mg mL^−1^).

### 3.6. Ln^3+^ Toxicity and Accumulation

**Figure 8:**
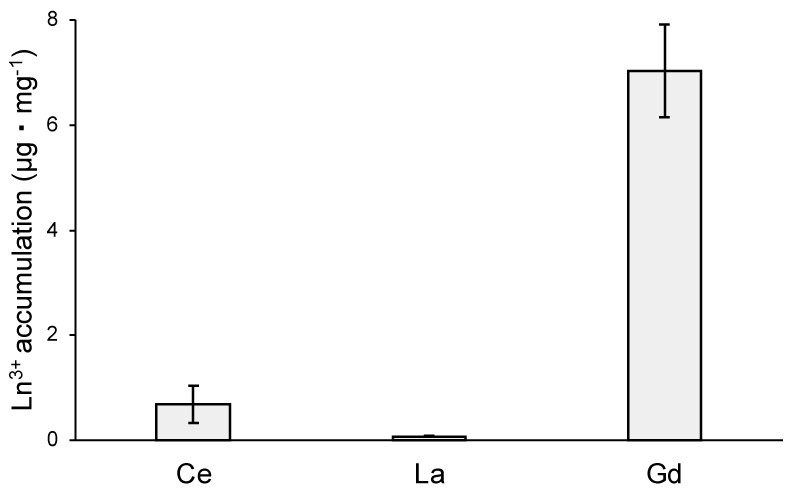
Ln^3+^ accumulation capability of *B. stratosphericus*, expressed as μg · mg^−1^ of dry weight.

MCML, due to its ability to solubilize high concentrations of Ln^3+^, may allow studying the different effects of interactions between Ln^3+^ and microorganisms. In this regard, the toxic effects and cellular accumulation of Ce^3+^, La^3+^, Gd^3+^ were evaluated for *B. stratosphericus* strain. This Gram-positive strain was previously selected for NaCl and heavy metal high tolerance, exopolysaccharide production, and resistance to high temperatures (up to 55 °C) [41,42]. The results showed a relation between dose and antimicrobial action for all tested Ln^3+^, as evidenced by a reduction in the number and diameter of colonies on agarized medium. At 10.0 mM, Ce^3+^ reduced the bacterial population by about 70%, while La^3+^ and Gd^3+^ by 90 and 97%, respectively (Table 4, Figure S4). Moreover, the colony diameters were reduced by about 77%, 55%, and 30% in the presence of 10 mM Ce^3+^, La^3+^, and Gd^3+^, respectively (Table 5, Figure S4). Finally, the capability of *B. stratosphericus* to accumulate Ln^3+^ was estimated. In particular, the results highlighted a greater accumulation capability for Gd^3+^ (Figure 8—7.04 ± 0.88 μg·mg^−1^ dry weight) compared to Ce^3+^ and La^3+^ (Figure 8—0.69 ± 0.36 μg·mg^−1^ and 0.076 ± 0.015 μg·mg^−1^, respectively).

## 4. Discussion

The use of minimal culture media is essential for physiological, metabolic, molecular, and genetic studies of microorganisms because it allows control of experimental conditions by reducing the multiple variables. Understanding cellular processes and their regulation is essential for basic and applied research. These studies allow us to control the growth of microorganisms or select and/or engineer strains useful for biotechnological applications such as productive processes (antibiotics, enzymes, drugs, and metal bioleaching) or contaminants removal (e.g., inorganics or organics) [44,45,46].

One of the main problems in studying the interaction between microorganisms and Ln^3+^ is the poor solubility of these elements and their consequent precipitation in the culture media, even at low concentrations, mainly caused by reactions with phosphate ions. To date, in the scientific literature, only a few research studies have faced this problem. Rasoulnia et al. (2022) developed a culture medium in which the yeast extract was employed as an alternative phosphate source to reduce precipitation [47]. However, yeast extract, due to its complex composition, makes the medium similar to a maximal medium and not suitable for minimal media purposes. Groom and Lidstrom (2021) developed a minimal culture medium to study the interaction of Ln^3+^ with microorganisms using citrate as a chelator [48]. This medium had a high pH (8.0–9.0) and was specific for haloalkaliphilic methanotroph bacteria; moreover, it was tested only with La^3+^ and up to a concentration of 30 μM, almost 35 times lower than those used in MCML.

Ene et al. (2015) showed the toxicity of Ln^3+^ on *Saccharomyces cerevisiae* using the minimal culture medium “MM” [49]. The authors highlighted the correlation between Ln^3+^ accumulation, both inside and on the cell surface, other than inhibition of Ca^2+^ uptake [50]. However, the Ln^3+^ concentration was not clearly defined, and precipitation problems were only alluded to. In other studies, to overcome the precipitation problems, distilled water, water saline solution, or acid conditions, in which these elements are more soluble, were used as analytical medium [51]. This strategy has been used to evaluate Ln^3+^ absorption by microbial cells, as described by Kazak et al. (2021). In this case, the absorption was estimated in water saline solutions (0.01 M NaCl), dissolving 1.0 mg·L^−1^ of Ln^3+^, and it was highlighted that the process is influenced by both pH and bacterial species [52]. However, the use of the above-mentioned solutions, where most nutrients are absent or insufficient, can significantly affect cell vitality, altering the metabolism and inducing stress responses, which overall lead to misleading results [53].

Unlike the above, in our experiment, the developed MCML allowed the solubilization of a large amount of Ln^3+^, from 1.0 mM (La^3+^, Ce^3+^) up to 5.0 mM (Gd^3+^) at a pH of 6.0–7.0; therefore, it is able to support the growth of many microorganisms of different taxa. Moreover, it was observed up to 100 times higher solubility of La^3+^, Ce^3+^, Gd^3+^ compared to common tested minimal media (DF, DM, CD).

In our experimental conditions, Ce^3+^ solubility in MCML (Table 3, Figure 3) did not agree with the predicted model obtained assuming a logK_s_ value of −26.27, as reported in the scientific literature for aged solids [data source: https://equilibriumdata.github.io/guide/ (accessed on 1 April 2024)]. To model the experimental observation, in the data set used to plot the distribution diagram (Figure 2), a logK_s_ of −19 had to be assumed for CePO_4_(s) freshly formed instead of the above-reported value for the aged solid. In our model, a solubility product for CePO_4_(s) that is about 7 orders of magnitude different from the one reported in the literature is the only way to describe our experimental data. It should be considered that the solubility products reported in the literature refer to thermodynamically stable solid phases, which are known to be by far more insoluble than the solids formed from supersaturated solutions. In addition, the formation constants available for the Ce(III)-citrate system are questionable, and the existence of complexes not considered in the model is quite probable. Based on these considerations, the model, though very useful, must be considered in some way approximate.

In MCML, the citrate buffer replaces the classic phosphate buffer present in many culture media in order to reduce the quantity of free phosphate, one of the main causes of Ln^3+^ precipitation in its salt-insoluble form [54]. Furthermore, citrate can complex metals, preventing their precipitation and increasing their solubility [55]. It is also a source of organic carbon and a growth factor for many microorganisms [56,57], although at high concentrations it is toxic [58]. The quantity of citrate solubilized in MCML (~11.0 g L^−1^) is lower than the reported toxicity values (~15.0 g L^−1^) [59], and microorganism growth confirms the tolerability of the concentration employed.

The MCML supports the growth of a high number of environmental microorganisms, both bacteria and fungi. The growth of 82 bacterial strains, belonging to more than 30 different morphological groups, as well as that of five fungi species, was tested. It was observed that MCML supports the growth of 63 bacterial strains (about 75% of tested ones) and 5 fungi compared to a rich medium (PCA), and these results were like those obtained for the other assayed minimal media (DM, CD, DF). It is noteworthy that MCML also supported the growth of *E. coli* and pathogenic *S. aureus* strains.

Among the known genera used in this work, only *Halomonas titanicae* did not grow on MCML. For the Halomonas genus, the citrate concentration used should not be toxic; in fact, Zhang et al. (2020) have observed excellent growth of *Halomonas* sp. (TDO1 strain) in the presence of citrate up to 30 g L^−1^ [60]. The lack of growth of *H. titanicae* could depend both on the absence of specific nutrients in the culture medium (macronutrients, micronutrients, and growth factors) and/or on its physiological characteristics. This bacterial specie is halophilic and requires appropriate concentrations of NaCl [61]; therefore, the growth in MCML might require the achievement of suitable osmotic pressure in the medium.

Afterward, the efficacy of MCML was quantified by studying the kinetic growth of *B. stratosphericus* and *D. hansenii*, representative of bacterial and fungal taxa, respectively. The growth parameters were compared with those of other common culture media, such as DM (for bacteria) or CD (for fungi). The *B. stratosphericus* strain highlighted a lag phase slightly longer in MCML compared to DM, but a better growth rate and ultimately greater biomass production. A similar trend was observed in the case of MCML for *D. hansenii* when compared with CD. This result could be due to the metabolic adaptation of both microorganisms to the different medium compositions, such as the presence of citrate and a lower phosphate concentration in MCML with respect to the reference media. On the other hand, the higher growth rate and amount of biomass produced might be due to the presence of citrate as a carbon source in addition to glucose or sucrose.

Finally, we verified the suitability of MCML for Ln^3+^ studies by conducting a preliminary evaluation of their biological effects on *B. stratosphericus*. Our data on toxicity indicated a significant dose-dependent antimicrobial action of all compounds at concentrations of 5–10 mM. Similar results were show. by Wakabayashi et al. (2016) for Ce^3+^, La^3+^, and Gd^3+^, highlighting the major toxicity in the case of La^3+^ [62]. However, in their study, the toxic effects were determined on Gram-negative *E. coli* using a rich medium (LB). Moreover, we evidenced the ability of *B. stratosphericus* to accumulate Ln^3+^ ions, in particular Gd^3+^. We did not investigate the nature of these interactions, absorption into envelope structures, or uptake into cells. In this regard, it is known that the *B. stratosphericus* strain produces exopolysaccharides (EPSs) [63], which are involved in biofilm formation and protection from stress conditions (e.g., contaminated and saline environments). EPSs have anionic groups that bind cations, such as heavy metals, Na^+^, K^+^, retaining and accumulating them on the cell surface. This mechanism reduces cellular availability and toxic effects, and similarly, it could also play a crucial role in the accumulation of Ln^3+^.

## 5. Conclusions

MCML developed and used in our study could fill the lack in the scientific literature of a minimal medium suitable for the cultivation of microorganisms in the presence of great Ln^3+^ concentrations. This also allows for an in-depth study of microorganism physiological processes crucial for the development of new relevant biotechnological applications. Furthermore, MCML can be defined as a broad-spectrum medium suitable for the screening of both many environmental microorganisms and those of human health relevance. Finally, the results of our study open up new considerations on the need for reliable equilibrium constants relative to the complexes of Ln^3+^ with phosphate, citrate, and other relevant organic ligands typical of the culture media.

## Figures and Tables

**Table 1:** Minimal culture medium composition.

Components	Concentration in Medium [g L^−1^]			
	MCML	DM	CD	DF
(NH_4_)_2_SO_4_	2.0	1.0	2.0 *	2.0
MgSO_4_·7H_2_O	0.2	0.1	0.5	0.2
KH_2_PO_4_	0.4	3.0	-	4.0
K_2_HPO_4_	-	7.0	1.0	-
Na_2_HPO_4_	-	-	-	6.0
KCl	-	-	0.5	-
Na_3_citOH·2H_2_O	10.02	0.5	-	-
H_3_citOH	0.94	-	-	2.0
Gluconic acid	-	-	-	2.0
FeSO_4_·7H_2_O	0.0001	-	0.01	0.0001
D-Glucose	2.0	2.0	-	2.0
Sucrose	-	-	30.0	-
L-arginine	-	0.02	-	-
L-tryptophan	-	0.02	-	-
Micronutrients	100 µL **	-	-	100 µL ***
pH	6.0	7.0	7.3	6.0

* NaNO_3_, the nitrogen source typically used in Czapek Dox, was replaced with (NH_4_)_2_SO_4_; ** Micronutrients are described in the MCML preparation paragraph; *** DF micronutrients are the same described in MCML.

**Table 2:** Solubility of different Ce^3+^ concentrations and pH values in MCML.

mM	pH						
	2.0	3.0	4.0	5.0	6.0	7.0	8.0
0.01	+	+	+	+	+	+	+
0.05	+	+	+	+	+	+	+
0.1	+	+	+	+	+	+	+
0.5	+	+	+	+	+	+	+
1.0	+	+	+	+	+	+	−
2.0	+	+	+	+	−	−	−
3.0	+	+	+	−	−	−	−
5.0	+	−	−	−	−	−	−

“+” indicates the Ce^3+^ was still in solution and no precipitation was observed; “−” indicates the presence of a precipitate on the bottom of the test-tube in the form of fluffy white cloud.

**Table 3:** Maximum solubility (mM) of La^3+^, Ce^3+^, and Gd^3+^ in different liquid culture media, evaluated at 25 °C and at respective pH values. “-” = Insoluble at the minimal tested concentration (0.01 mM).

Culture Medium	pH	La^3+^	Ce^3+^	Gd^3+^
MCML	6.0	1.0	1.0	5.0
Dworkin and Foster	6.0	0.01	0.01	0.05
Davis and Mingioli	7.0	0.05	0.01	0.05
Czapek Dox	7.3	-	-	-

**Table 4:** *B. stratosphericus* colony number for each Ln^3+^ treatment at different concentrations (mM). “ND” = not determined; “-” = absence of growth.

Ln^3+^ (mM)	Colony Numbers in the Follow Ln^3+^ Treatments		
	Ce^3+^	La^3+^	Gd^3+^
0 (Control)	166 × 10^5^	166 × 10^5^	166 × 10^5^
5	121 × 10^5^	900 × 10^4^	ND
10	48 ×10^5^	147 × 10^4^	54 × 10^4^
20	ND	ND	-

**Table 5:** Colony diameters (mm) of *B. stratosphericus* measured with ImageJ software, version 1.53t, for each Ln^3+^ treatments at different concentrations (mM). Letters a, b, c represents the significant statistical differences in the same treatment at increasing concentration; “ND” = not determined; “-” = absence of growth.

Ln^3+^ (mM)	Colony Diameter (mm) in the Follow Ln^3+^ Treatments		
	Ce^3+^	La^3+^	Gd^3+^
0 (Control)	2.14 ± 0.17 a	2.17 ± 0.47 a	2.05 ± 0.17 a
5	0.89 ± 0.15 b	0.87 ± 0.17 b	ND
10	0.49 ± 0.08 c	0.99 ± 0.12 b	1.43 ± 0.22 b
20	ND	ND	-

## Data Availability

The authors declare that the data supporting the findings of this study are available within the paper and its Supplementary Materials. Should any raw data files be needed in another format they are available from the corresponding author upon reasonable request. Source data are provided with this paper.

## Supplementary Materials

The following supporting information can be downloaded at: https://www.mdpi.com/article/10.3390/microorganisms12081531/s1. Figure S1: Distribution diagram in the presence of 3.0 mM total phosphate up to pH 1.35, when the formation of solid CePO_4_ occurs; Figure S2: Distribution diagram of 1.0 mM Ce(III) in the presence of 40.0 mM total citrate; Figure S3: Bacterial cultivation on different agarized culture media after 120 h (5 days) of incubation; Figure S4: Ln^3+^ toxicity test. (A), Control; (B), Ce^3+^ 5 mM; (C), Ce^3+^ 10 mM; (D), La^3+^ 5 mM; (E), La^3+^ 10 mM; (F), Gd^3+^ 10 mM; (G), Gd^3+^ 20 mM; Table S1: Equilibrium constants used in the development of Ce(III) speciation models at 25 °C in the MCML soil. Data source: https://equilibriumdata.github.io/guide/ (access: January 2024). H_3_citOH indicates the fully protonated citric acid; Table S2. Microbial growth on different culture media after five days of incubation. “+” indicates that the microorganism was growth, “-” indicates absence of growth; “n.e.” = not evaluated. Gram = Gram’s stain; PCA = Plant Count Agar; MCML = Minimal Culture Medium for Lanthanides; DM = Davis Mingioli; CD_s30_ = Czapek Dox with 30 gL^-1^ sucrose as carbon source; CD_s2_ = Czapek Dox with 2.0 gL^-1^ sucrose as carbon source; DF = Dworkin Foster
