# Supplementary material for: Development of Broad-Range Microbial Minimal Culture Medium for Lanthanide Studies
Source: Microorganisms. 2024 Jul 26;12(8):1531. doi: 10.3390/microorganisms12081531 (PMC11356471; doi:10.3390/microorganisms12081531)
Supplement: Supplementary file 1 [file microorganisms-12-01531-s001.zip › microorganisms-3103836-supplementary.pdf]

Supplementary Material - Development of Broad-Range Microbial Minimal Culture Medium for Lanthanide Studies

Gianmaria Oliva[1], Giovanni Vigliotta[1*], Luca Di Stasio[1], Ermanno Vasca[1], Stefano Castiglione[1]

[1] Department of Chemistry and Biology "A. Zambelli", University of Salerno, 84084 Fisciano (SA), Italy

*** Correspondence:**
Corresponding Author
gvigliotta@unisa.it

Table S1. Equilibrium constants used in the development of Ce(III) speciation models at 25 °C in the MCML soil. Data source: https://equilibriumdata.github.io/guide/ (access: January 2024). H_3citOH indicates the fully protonated citric acid

**Reaction**	**log(constant)**
$H_2O = H^+ + OH^-$	-14.00
$H_3PO_4 = H^+ + H_2PO_4^-$	- 2.12
$H_2PO_4^- = H^+ + HPO_4^{2-}$	- 7.20
$HPO_4^{2-} = H^+ + PO_4^{3-}$	- 12.33
$H_3citOH = H^+ + H_2citOH^-$	- 3.13
$H_2citOH^- = H^+ + HcitOH^{2-}$	- 4.76
$HcitOH^{2-} = H^+ + citOH^{3-}$	- 6.40
$citOH^{3-} = H^+ + citO^{4-}$	- 14.4
$Ce^{3+} + H_2O = CeOH^{2+} + H^+$	- 8.1
$Ce^{3+} + 2H_2O = Ce(OH)^{2+} + 2H^+$	- 16.3
$Ce^{3+} + 3H_2O = Ce(OH)_3 + 3H^+$	- 26.0
$3Ce^{3+} + 5H_2O = Ce_3(OH)_5^{4+} + 5H^+$	- 32.8
$Ce(OH)_3(s) + 3H^+ = Ce^{3+} + 3H_2O$	- 21.2
$CePO_4(s) = Ce^{3+} + PO_4^{3-}$	- 26.27
$Ce^{3+} + H^+ + citOH^{3-} = Ce(HcitOH)^+$	11.5
$Ce^{3+} + citOH^{3-} = Ce(citOH)$	6.7
$Ce^{3+} + 2H^+ + 2citOH^{3-} = Ce(HcitOH)_2^-$	20.74
$Ce^{3+} + 2citOH^{3-} = Ce(citOH)_2^{3-}$	11.21

Table S2. Microbial growth on different culture media after five days of incubation.
"+" indicates that the microorganism was growth, "-" indicates absence of growth; "n.e." = not evaluated. Gram = Gram's stain; PCA = Plant Count Agar; MCML = Minimal Culture Medium for Lanthanides; DM = Davis Mingioli; CD_{s30} = Czapek Dox with 30 gL^{-1} sucrose as carbon source; CD_{s2} = Czapek Dox with 2.0 gL^{-1} sucrose as carbon source; DF = Dworkin Foster

**Strain**	**Gram**	**Culture media**					
		**PCA**	**MCML**	**DM**	**CD_{s30}**	**CD_{s2}**	**DF**
			Fungi				
S. cerevisiae (yeast)	n.e.	+	+	+	+	n.e.	+
D. hansenii (yeast)	n.e.	+	+	+	+	n.e.	+
Sclerotium sp. (mold)	n.e.	+	+	+	+	n.e.	+
AL18 (mold)	n.e.	+	+	+	+	n.e.	+
DO24 (mold)	n.e.	+	+	+	+	n.e.	+
			Bacteria				
E. coli	-	+	+	-	+	+	+
S. aureus	+	+	+	-	-	-	-
B. stratosphericus	+	+	+	+	+	+	+
H. titanicae	-	+	-	+	-	-	-
H. alkaliphila	-	+	+	+	-	-	+
3RR23	+	+	-	-	+	+	-
1RS2	-	+	+	-	+	+	+
1TS27	-	+	+	+	+	+	+
3TR25	-	+	+	-	+	+	+
2TS9	-	+	+	-	+	+	+
1TR41	-	+	+	-	+	+	+
3TS19	+	+	-	-	+	+	-
2RR12	-	+	+	-	+	+	+
3RR18	-	+	+	-	+	+	+
3TR27	+	+	+	-	+	+	+
2RS7	+	+	-	-	+	+	-
1TS15	+	+	+	+	+	+	+
2TS10	+	+	+	+	+	+	+
2RR13	-	+	+	+	+	+	+
3TS12	+	+	-	-	+	+	-
1RR10	-	+	+	+	+	+	+
3RR40	-	+	+	-	+	+	+
3TR28	-	+	+	-	+	+	+
2RS1	+	+	+	+	+	+	+
2TS16	+	+	+	-	+	+	+

2RR5	+	+	+	+	+	+	+
3TS13	+	+	-	-	+	+	+
1TS22	-	+	+	+	+	+	+
3RR16	-	+	+	-	+	+	+
3TR55	+	+	+	+	+	+	+
3TS1	+	+	-	-	+	+	-
2RS29	-	+	-	+	+	+	-
2RS15	-	+	+	+	+	+	-
2RR21	-	+	+	-	+	+	+
3TS15	-	+	-	+	+	+	+
2TS24	-	+	-	-	+	+	-
3RS5	+	+	+	-	+	+	+
3TR56	-	+	+	+	+	+	+
3TS6	+	+	+	+	+	+	+
2TS20	-	+	+	-	+	+	+
2RS21	-	+	-	+	+	+	+
2TR7	+	+	-	-	+	+	+
1TR2	+	+	-	-	+	+	-
1TS4	+	+	+	+	+	+	+
2TS25	+	+	+	+	+	+	+
1TR11	+	+	-	-	+	+	+
3RS7	+	+	+	+	+	+	+
1RS24	-	+	-	-	+	+	+
2RS23	-	+	-	-	+	+	-
2TR4	+	+	+	+	+	+	+
1RR26	-	+	-	-	+	+	-
2TR21	+	+	+	-	+	+	+
3TS26	-	+	+	+	+	+	+
1TR14	-	+	+	+	+	+	+
3RS6	+	+	+	+	+	+	+
1TS2	+	+	+	+	+	+	+
2RS26	-	+	+	+	+	+	+
1TR17	-	+	+	-	+	+	+
3TR13	-	+	+	+	+	+	+
1TS25	-	+	+	+	+	+	+
2TS26	+	+	+	+	+	+	+
1TR8	+	+	+	+	+	+	+
3RS15	+	+	-	-	+	+	-
3RR10	+	+	-	-	+	+	-

3RR1	-	+	+	-	+	+	+
1TR16	+	+	+	-	+	+	+
3TR19	-	+	+	+	+	-	+
2TS6	-	+	+	+	+	+	+
1RS1	-	+	+	+	+	+	+
1TR15	-	+	+	+	+	+	+
3TR17	+	+	+	+	+	+	+
3RR9	-	+	-	-	+	+	+
2TR22	+	+	+	-	+	+	+
1TR25	-	+	+	-	+	+	+
3TR3	+	+	+	+	+	-	+
1RS6	-	+	+	-	+	+	+
1RS21	-	+	+	+	+	+	+
2RS14	-	+	-	-	+	-	-
3TR23	-	+	+	-	+	+	+
3RR6	+	+	+	+	+	+	+
3TR29	-	+	+	+	+	+	+
1TR21	-	+	+	-	+	+	+
3TR14	-	+	+	+	+	+	+
1RR13	-	+	+	+	+	+	+

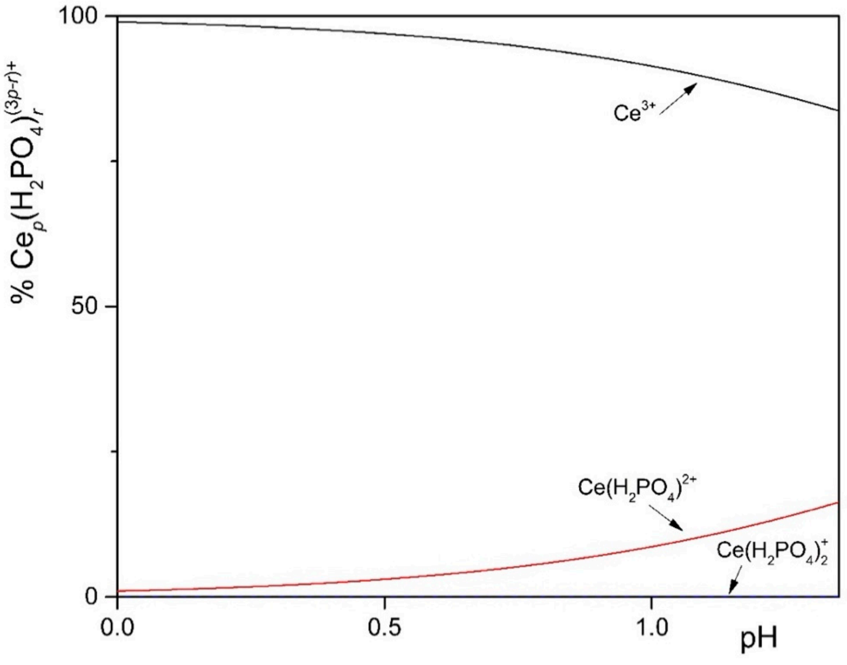

Figure S1. Distribution diagram in the presence of 3.0 mM total phosphate up to pH 1.35, when the formation of solid $CePO_4$ occurs

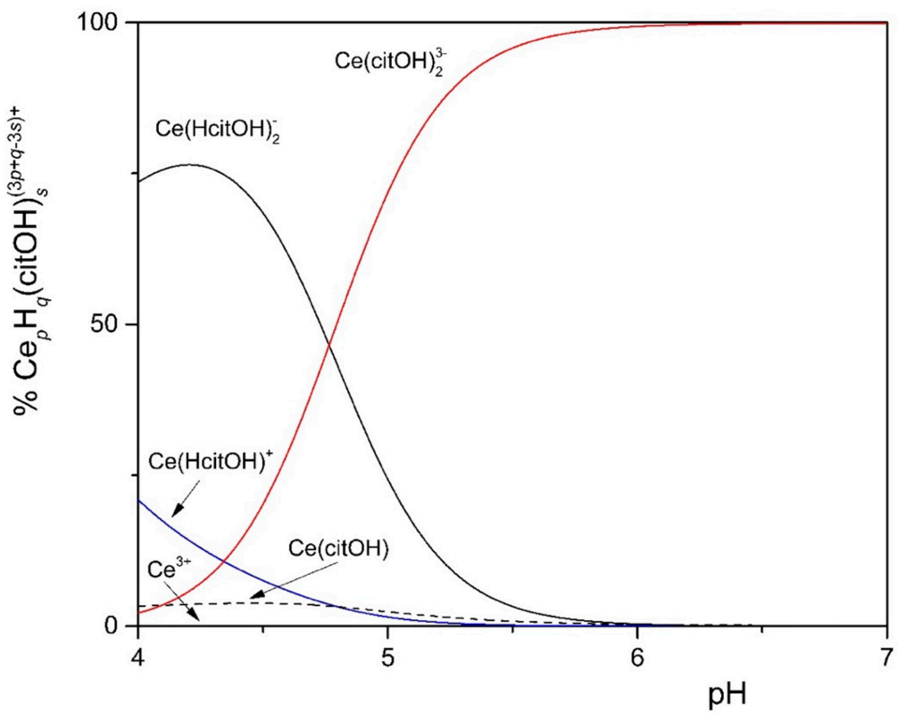

Figure S2. Distribution diagram of 1.0 mM Ce(III) in the presence of 40.0 mM total citrate

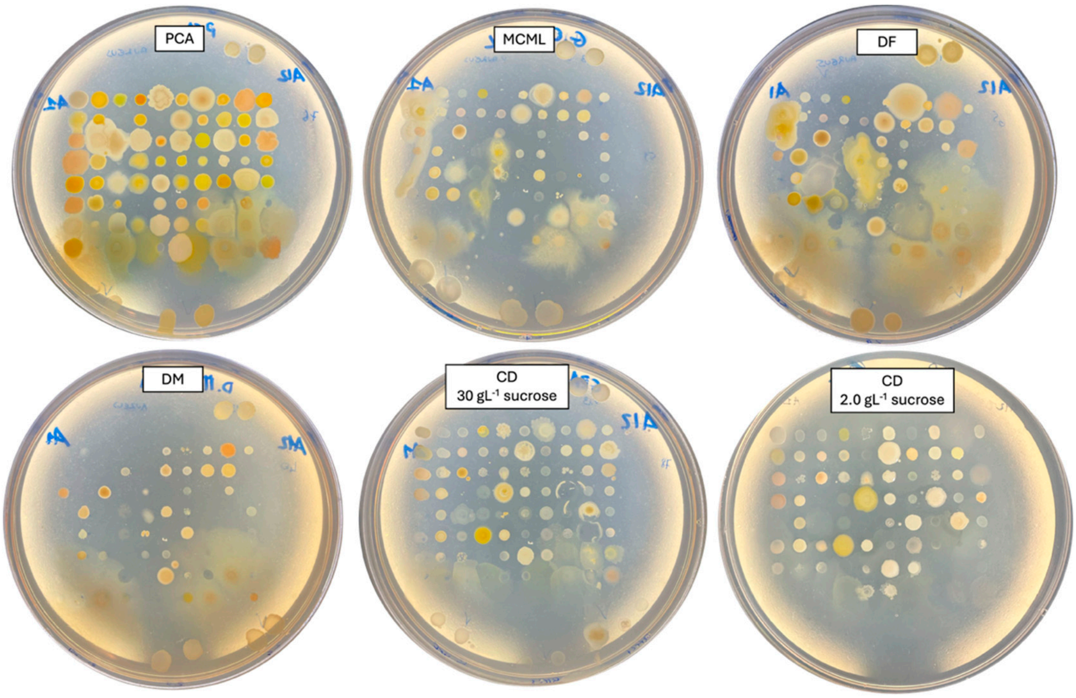

Figure S3. Bacterial cultivation on different agarized culture media after 120 h (5 days) of incubation

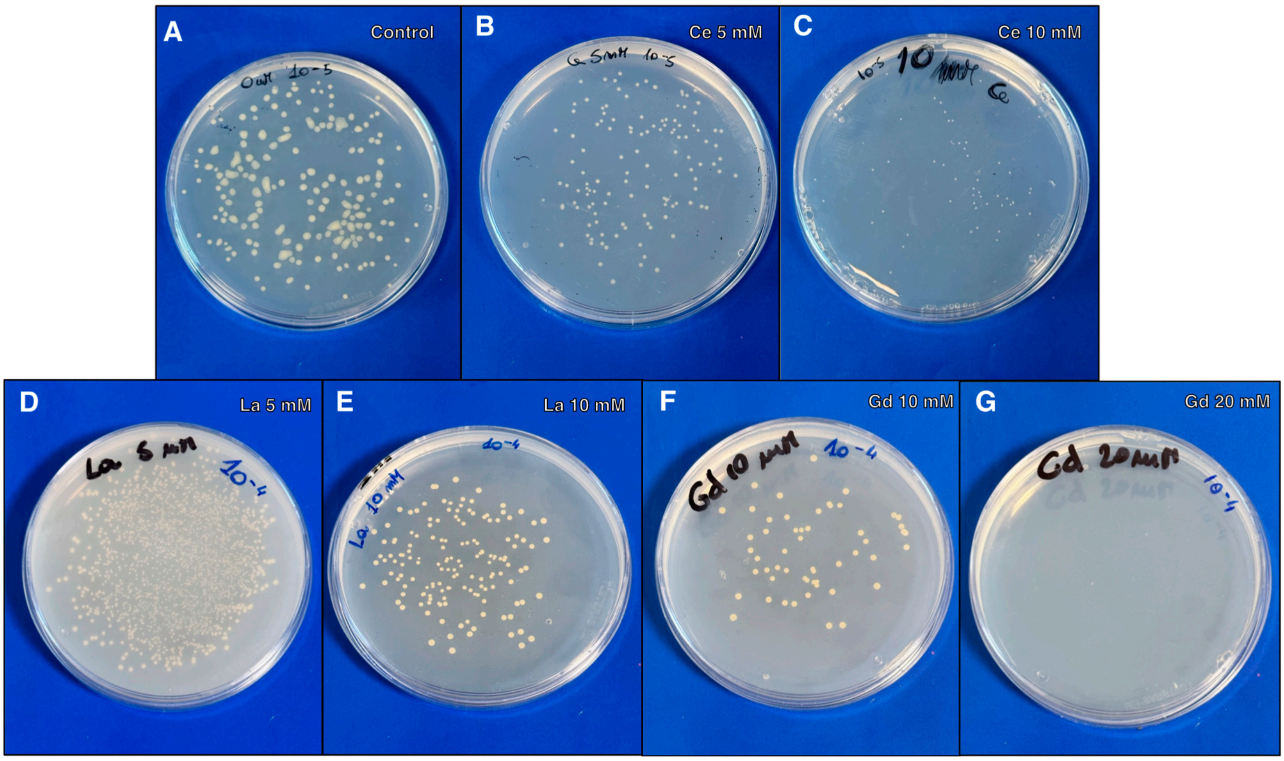

Figure S4. Ln^{3+} toxicity test. (A), Control; (B), Ce^{3+} 5 mM; (C), Ce^{3+} 10 mM; (D), La^{3+} 5 mM; (E), La^{3+} 10 mM; (F), Gd^{3+} 10 mM; (G), Gd^{3+} 20 mM
